# Reduced toxicity of malachite green decolorized by laccase produced from *Ganoderma* sp. rckk-02 under solid-state fermentation

**DOI:** 10.1007/s13205-014-0258-1

**Published:** 2014-11-11

**Authors:** Abha Sharma, Bhuvnesh Shrivastava, Ramesh Chander Kuhad

**Affiliations:** Lignocellulose Biotechnology Laboratory, Department of Microbiology, University of Delhi South Campus, Benito Juarez Road, New Delhi, 110021 India

**Keywords:** Decolorization, Detoxification, *Ganoderma* sp. rckk-02, Laccase, Malachite green

## Abstract

Statistical designs were applied for optimizing laccase production from a white-rot fungus, *Ganoderma* sp. rckk-02 under solid-state fermentation (SSF). Compared to unoptimized conditions [2,154 U/gds (Unit per gram of dry substrate)], the optimization process resulted in a 17.3-fold increase in laccase production (37,423 U/gds). The laccase produced was evaluated for its potential to decolorize a recalcitrant synthetic dye, malachite green. Laccase at dosage of 30 U/ml in presence of 1 mM of 1-hydroxybenzotriazole (HBT) almost completely decolorized 100 and 200 mg/l of malachite green in 16 and 20 h, respectively, at 30 °C, pH 5.5 and 150 rpm. While, higher dyes concentrations of 300, 400 and 500 mg/l were decolorized to 72, 62 and 55 % in 24, 28 and 32 h, respectively, under similar conditions. Furthermore, it was observed that the decolorized malachite green was less toxic towards the growth of five white-rot fungi tested viz. *Crinipellis* sp. RCK-1, *Ganoderma* sp. rckk-02, *Coriolopsis Caperata* RCK 2011, *Phanerochaete chrysosporium* K3 and *Pycnoporous cinnabarinus* PB. The present study demonstrates the potential of *Ganoderma* sp. rckk-02 to produce high titres of laccase under SSF, which can be exploited in conjunction with redox mediator for the decolorization of high concentrations of malachite green from water bodies.

## Introduction

Malachite green is a synthetic dye used extensively in aquaculture as a parasiticide/fungicide against protozoan and fungal infections in farmed fish (Maalej-Kammoun et al. 2009). In textile and leather industry, it is used for dying silk, wool, jute and leathers (Gupta et al. 2000). However, the use of this dye generates a lot of concern because of its known toxic effects, including organ damage, mutation and developmental abnormalities in mammals (Gouranchat 2000). The potential human exposure to the dye can occur by consumption of treated fish and by working in dye and aquaculture industry (Cha et al. 2001). The dye also being toxic to micro-organisms affects aquatic ecosystems in an adverse manner (Srivastava et al. 2004). Moreover, the toxic effects of malachite green increase with exposure to time, temperature and concentration as the dye reduces to leuco-malachite green (Leuco-MG), whose elimination rate is very slow (Srivastava et al. 2004; Papinutti et al. 2006). Hence, ways to remove excess/residual malachite green from treatment ponds and industrial effluents need to be explored.

Abiotic methods of dye reduction are known, but their implementation requires expensive catalysts and reagents, which themselves are not environmentally benign (Kuhad et al. 2004). While, use of micro-organisms and the enzymes they secrete is the best route forward for elimination of recalcitrant dyes in an eco-friendly manner (Diwaniyan et al. 2010). Among micro-organisms, white-rot fungi (WRF), group of lignin-degrading basidiomycetous fungi are very efficient in breaking down synthetic dyes as the structure of these dyes is similar to the components that make lignin content in wood (Diwaniyan et al. 2010). However, the microbiological biodegradation of malachite green is difficult because of its fungicidal nature (Maalej-Kammoun et al. 2009), thereby preventing implementation of in situ bioremediation strategies for removal of this dye. Alternatively, the isolated ligninolytic enzymes of white-rot fungi can be applied for combating this pollutant as the potential of WRF to degrade lignin and related compounds is due to their extracellular ligninolytic enzyme system comprising of laccase, lignin peroxidase and manganese peroxidase (Murugesan et al. 2007). Out of the three major ligninolytic enzymes produced by WRF, laccases are the most promising ones for various industrial and environmental applications. It is because these enzymes use atmospheric oxygen and release water as the sole by-product and do not require any cofactor or H_2_O_2_ like peroxidases do (Kidwai et al. 2013). Laccase is a polyphenol oxidase that oxidizes polyphneols, methoxy-substituted polyphenols and diamines using the distinctive redox ability of copper ions with the concomitant reduction of molecular oxygen to water (Thurston 1994). However, a major limitation for the commercialization of laccase-based processes is their low production levels and eventually the high cost. Therefore, it is imperative to produce high titres of laccase at low cost, which can be achieved by optimizing culture and fermentation conditions. In this regard, the use of solid-state fermentation (SSF) for enzyme production provides significant economic (Osma et al. 2011) and technical benefits including, high product yields (Kamra and Satyanaryana 2004; Mazumder et al. 2009), use of simple machinery, lesser generation of effluents and lower requirements for agitation (Szendefy et al. 2006). Moreover, the application of statistical designs such as Plackett–Burman design (PBD) for screening of factors that have greatest influence on enzyme production followed by response surface methodology (RSM) for defining their optimum levels is very useful in maximizing enzyme production. Keeping all this in view, the present work was aimed at enhancing laccase production from white-rot fungus *Ganoderma* sp. rckk-02 by optimizing culture conditions in SSF using statistical designs. The enzyme produced was evaluated for its potential to decolorize malachite green. The decolorized dye was further tested for its reduced toxicity against the growth of some white-rot fungi.

## Materials and methods

### Chemicals and raw materials

All assay reagents were purchased from Sigma-Aldrich (St. Louis, MO, USA), while all media components and dye malachite green were purchased from HiMedia Laboratories Pvt. Ltd. (Mumbai, India). Chemicals used were purchased from Fischer Scientific (Waltham, USA). Wheat bran was obtained locally.

### Micro-organisms and culture conditions

*P. cinnabarinus* PB and *P. chrysosporium* K3 were kindly gifted by Dr. K.-E.L Eriksson, Professor Emeritus, Department of Biochemistry and Molecular Biology, University of Georgia, Athens, USA. While, *Crinipellis* sp. RCK-1 (AM055944), *Ganoderma* sp. rckk-02 (AJ749970) and *Coriolopsis Caperata* RCK 2011 (JF283779) were procured from Culture bank, Lignocellulose Biotechnology Laboratory, Department of Microbiology, University of Delhi South Campus. The fungal isolates were grown and maintained on malt extract agar (MEA) containing (g/L): Malt extract 20, KH_2_PO_4_ 0.5, Ca(NO_3_)_2_·4H_2_O 0.5, MgSO_4_·7H_2_O 0.5, agar, 20 (pH 5.5.) as described earlier (Diwaniyan et al. 2010). Pure fungal cultures were stored at 4 °C and subcultured every fortnight.

### Laccase production from *Ganoderma* sp. rckk-02

SSF for laccase production was carried out in 250-ml Erlenmeyer flasks containing 5.0 g of wheat bran moistened with mineral salt solution containing g/l: Ca(NO_3_)_2_·4H_2_O, 0.5; KH_2_PO_4_, 0.5; MgSO_4_·7H_2_O in solid substrate to moisture ratio of 1:3 as described elsewhere (Sharma et al. 2005). The flasks were inoculated with desired volume of 7-day-old crushed fungal mat (equal to 0.25/5 g of substrate), mixed properly under aseptic conditions and kept at 30 °C. The flasks were patted gently at their bottoms to shake the substrate for air exchange at regular intervals of 24 h after the onset of fungal mycelial growth. To study the time course of laccase production from the fungus, the fungal fermented bran was removed from the flasks at regular intervals, suspended in citrate buffer (pH 5.5, 100 mM) in fermented solid to liquid ratio of 1:10 and shaken gently for 30 min at 30 °C. The extrudates were squeezed through muslin cloth for maximizing enzyme extraction and centrifuged at 10,000 rpm at 4 °C for 10 min. The enzyme extract thus obtained was assayed for laccase activity.

### Screening of variables using PBD

Screening of cultural and nutritional parameters (temperature, pH, moisture, inocula size, tryptophan, guaiacol, calcium nitrate, biotin and copper sulfate) influencing laccase production under SSF was carried out employing PBD. This design evaluates the relative importance of various parameters assuming there are no interactions between the factors. Based on the number of variables, experimental design matrix was constructed and analyzed using statistical software Design Expert 6.0 (Stat-Ease, Inc. Minneapolis). In the design matrix, each row represents an experiment and each column represents an independent variable, whose levels were varied (Table 1). A total number of *n* + 1 experiments were carried out, where *n* is the number of variables in study. The main effect (*E*_xi_) of any individual variable is calculated by the difference between the average of response at the high level (+1) and low level (−1). A large main effect (positive or negative) indicates that the variable has a larger impact on the response, while its value close to zero indicates that it does not have any significant effect on the response (Levin et al. 2008).

**Table 1:** Plackett–Burman design for laccase production from *Ganoderma* sp. rckk-02

Run	pH	Temp. (°C)	Moisture	Tryptophan (% w/w)	Guaiacol (% w/w)	Calcium nitrate (% w/v)	Inocula size (%v/w)	Inocula age (days)	Biotin (% w/w)	Copper sulfate (mM)	D1	D2	Production (U/gds)
1	8	28	4	0.1	2	0.2	20	10	0.1	0.5	1	−1	5,350
2	8	40	2	0.1	0.5	0.2	20	20	1	0.5	−1	1	920
3	4	40	4	0.1	0.5	0.02	20	10	1	2	1	−1	6,400
4	8	28	4	1	0.5	0.02	5	20	1	0.5	1	1	10,710
5	8	40	2	0.1	2	0.02	5	20	0.1	2	1	1	4,650
6	8	40	4	1	0.5	0.2	5	10	0.1	2	−1	−1	11,885
7	4	40	4	1	2	0.02	20	20	0.1	0.5	−1	1	11,850
8	4	28	4	0.1	2	0.2	5	20	1	2	−1	1	4,640
9	4	28	2	1	0.5	0.2	20	20	0.1	2	1	1	14,335
10	8	28	2	1	2	0.02	20	10	1	2	−1	−1	7,000
11	4	40	2	1	2	0.2	5	10	1	0.5	1	−1	12,225
12	4	28	2	0.1	0.5	0.02	5	10	0.1	0.5	−1	−1	3,990

#### Optimization of screened variables using RSM

The significant variables identified by PBD were further optimized by RSM employing a central composite design (CCD). The chosen variables were analyzed at five different levels (−*α*, −1, 0, +1, +*α*) (Table 2) and different experimental combinations in a total of 20 standard runs (Table 3). The results obtained after CCD were analyzed through standard analysis of variance (ANOVA) and the behavior of the model in terms of mathematical relationship for laccase production was explained using second-order polynomial equation.

1 $$Y = \beta_{0} + \beta_{1} A + \beta_{2} B + \beta_{3} C + \beta_{11} A^{2} + \beta_{22} B^{2} + \beta_{33} C^{2} + \beta_{12} AB + \beta_{23} BC + \beta_{13} AC$$

where, *Y* is predicted response; *β*_0_ is intercept; *β*_1_, *β*_2,_ *β*_3_ are linear coefficients; *β*_11_, *β*_22_, *β*_33_ are squared coefficients and *β*_12_, *β*_23_, *β*_13_ are interaction coefficients.

**Table 2:** Experimental range and levels of independent variables studied by CCD in terms of actual and coded factors

Variable	Units	Coded value	Level of variable				
			−*α*	−1	0	+1	+*α*
Moisture	S:L ratio	*A*	1.65	2.5	3.75	5.0	5.85
Tryptophan	% w/w	*B*	0.98	2.5	3.75	5.0	6.02
Copper	mM	*C*	0.48	1.5	3.0	4.5	5.52

**Table 3:** Experimental design and results of central composite design of response surface methodology using three independent variables

Run	Moisture	Tryptophan (%w/w)	Copper (mM)	Laccase production (U/gds)	
				Experimental	Predicted
1	2.5	2.0	1.5	12,354.11	12,149.67
2	5.0	2.0	1.5	7,562.09	7,829.82
3	2.5	5.0	1.5	17,899.56	18,322.00
4	5.0	5.0	1.5	23,467.24	23,701.66
5	2.5	2.0	4.5	37,112.15	37,423.36
6	5.0	2.0	4.5	15,402.38	15,525.01
7	2.5	5.0	4.5	23,456.00	23,734.19
8	5.0	5.0	4.5	10,785.76	11,535.35
9	1.65	3.5	3.0	17,709.01	17,491.66
10	5.85	3.5	3.0	4,156.87	3,601.15
11	3.75	0.98	3.0	24,103.12	24,070.21
12	3.75	6.02	3.0	26,645.15	25,905.61
13	3.75	3.5	0.48	15,767.66	15,601.43
14	3.75	3.5	5.52	27,230.19	26,623.38
15	3.75	3.5	3.0	20,782.46	20,606.75
16	3.75	3.5	3.0	20,050.84	20,606.75
17	3.75	3.5	3.0	20,476.00	20,606.75
18	3.75	3.5	3.0	20,980.15	20,606.75
19	3.75	3.5	3.0	20,657.64	20,606.75
20	3.75	3.5	3.0	20,563.00	20,606.75

Fisher’s test was employed for checking the statistical significance of the equation. The quality of the fit of the equation was determined by coefficient of determination (*R* ^2^) and the adequacy of the model was checked by plotting a normal probability plot.

### Estimation of laccase activity

Laccase activity was estimated using guaiacol as substrate as described elsewhere (Diwaniyan et al. 2010). A change in absorbance of 0.01 min^−1^ ml^−1^ at 470 nm was defined as one unit of laccase activity (U).

### Decolorization of malachite green by laccase from *Ganoderma* sp. rckk-02

#### Effect of mediator on decolorization of malachite green by laccase

To study the effect of mediator on decolorization of malachite green by laccase, 20 U/ml of the enzyme was added to 50.0 ml of the dye solution (100 mg/l, pH 5.5) with and without 1 mM HBT and kept at 30 °C, 150 rpm for 36 h. Decolorization of Malachite green was recorded spectrophotometrically at 619 nm (*λ*_max_ for malachite green) using a UV–vis spectrophotometer (Analytik-Jena Specord 205). The control sample containing citrate phosphate buffer (pH 5.5) in place of enzyme was run in parallel. The percentage of decolorization was calculated as follows:

$${\text{Percentage of decolorization }}\left( \% \right){\text{ }} = {\text{ }}\left( {A_{{\text{c}}} - A_{{\text{t}}} } \right)/A_{{\text{c}}} \; \times \;100$$

where, *A*_c_ is the absorbance of the control and, *A*_t_ is the absorbance of the test sample.

#### Effect of enzyme dose on decolorization of malachite green by laccase

The effect of laccase dose on decolorization of malachite green was studied by incubating 100 mg/l of dye solution (pH 5.5) with different enzyme activity levels (10–40 U/ml) at 30 °C and 150 rpm. Percentage decolorization was measured in regular intervals of 4 h for 36 h.

#### Effect of dye concentration on decolorization of malachite green by laccase

The effect of malachite green concentration on its decolorization by laccase was studied at different dye concentrations ranging from 100 to 500 mg/l. Reaction mixtures (pH 5.5) containing different dye concentrations were incubated with 30 U/ml of laccase at 30 °C and 150 rpm. Percentage decolorization was measured in regular intervals of 4 h for 36 h.

### Evaluation of malachite green toxicity towards fungi

Five fungal isolates namely, *Crinipellis* sp. RCK -1, *C. caperata* RCK 2011, *Ganoderma* sp. rckk-02, *P. chrysosporium* K3 and *P. cinnabarinus* PB were grown on MEA amended with different concentrations of malachite green (100–500 mg/l) and incubated at 30 °C for 7 days. Control experiments in which fungal isolates were grown on MEA were also run in parallel. Radial growth in all the plates was measured at four positions from the point of inoculation and average was taken.

### Fungal viability on decolorized malachite green

Malachite green at concentrations ranging from 100 to 500 mg/l was pre-treated with 30 U/ml of laccase in presence of 1 mM HBT for 16, 20, 24, 28 and 32 h, respectively, and incorporated into MEA medium. All the fungal cultures were grown on this media and incubated at 30 °C for 7 days. Radial growth in all the plates was measured at four positions from the point of inoculation and average was taken.

## Results and discussion

### Laccase production from *Ganoderma* sp. rckk-02 under SSF

**Fig. 1:**
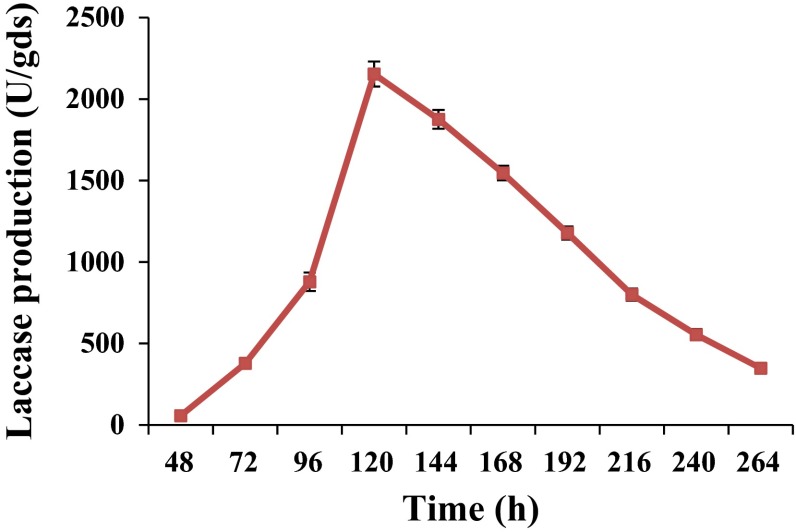
Time course of laccase production from *Ganoderma* sp. rckk-02

*Ganoderma* sp. rckk-02 exhibited laccase production after 48 h of incubation which reached maximum levels [2,154 ± 132.56 U/gds (unit per gram of dry substrate)] after 120 h of incubation and declined thereafter (Fig. 1).

### Optimization of laccase production from *Ganoderma* sp. rckk-02 using statistical designs

#### Screening of critical variables using PBD

Among the nine variables tested using PBD, tryptophan, copper and moisture content were significant factors affecting laccase production under SSF with *P* < 0.0001 (Table 4). While studying the effect of each factor, tryptophan emerged as the most significant factor by showing highest positive effect (7,009), which was followed by the effect of copper (2,230) and moisture content (1,285) (Table 4). Hence, these three factors viz. moisture (A), tryptophan (B) and copper (C) were selected for further optimization of their levels for laccase production from *Ganoderma* sp. rckk-02 under SSF by RSM using CCD. A huge difference in laccase production was observed in PBD experiments, showing the necessity of optimization of variables for enhancing enzyme production (Table 1).

**Table 4:** ANOVA for Plackett–burman design

Variable	Effect	*P* value (Prob > *F*)
pH	−2,154	0.008
Temperature	317	0.049
Moisture	1,285	<0.0001
Tryptophan	7,009.17	<0.0001
Guaiacol	−420.83	0.092
Calcium nitrate	792.50	0.112
Inocula size	−374.17	0.143
Inocula age	42.50	0.067
Biotin	−1,694.17	0.004
Copper	2,230.833	<0.0001

#### Optimization of screened variables by RSM of CCD

The optimum levels of selected factors (moisture, tryptophan and copper) for maximum enzyme production and the effect of their interactions on the response were determined using RSM of CCD in a set of 20 runs (Table 3). Maximum laccase production (37,423.36 U/gds) was predicted by the model when the fungus was grown on wheat bran moistened with mineral salt solution in substrate to moisture ratio of 1:2.5 supplemented with 4.5 mM copper and 2.0 % w/w tryptophan (Table 3).

#### Analysis of variance (ANOVA) for response surface model

The data obtained by RSM were analyzed by ANOVA (Table 5) which gave second-order regression Eq. 2 as a function of initial values of variables for laccase production from *Ganoderma* sp. rckk-02:

2 $$Y = 20606.75 - 4129.67A + 545.67B + 3,276.84C - 3556.87A^{2} + 1548.97 \, B^{2} + 178.78 \, C^{2} + 2424.88 \, AB - 4394.63 \, AC - 4965.38 \, BC$$

where laccase production (*Y*) is a function of moisture (*A*), tryptophan (*B*) and copper (*C*).

**Table 5:** Analysis of variance (ANOVA) for response surface model for laccase production

Term	Value
*F* value*	390.56
*P* > F**	<0.0001
*R* ^2#^	0.9972
Adj *R* ^2^	0.9946
Pred *R* ^2^	0.9812
Coefficient of variance	2.78
Adequate precision	88.825

* The computed *F* value of 390.56 indicates that there is only a 0.01 % chance that such high-model *F* value occurs due to noise

** According to the present model, the model terms *A*, *B*, *C*, *A* ^2^, *B* ^2^, *AB*, *AC* and *BC* were significant for laccase production exhibiting confidence level above 95 % (*P* > *F* < 0.05)

# The determination of coefficient (*R* ^2^) was 0.9972, explaining 99.72 % variability in the response

**Fig. 2:**
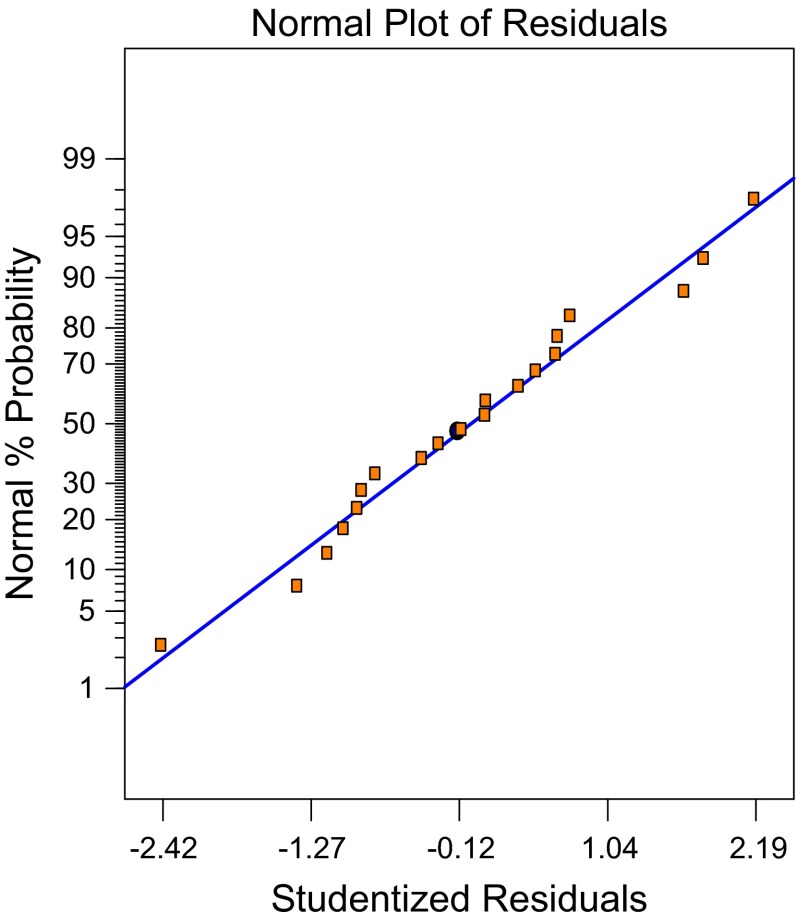
Residual plot for laccase production

 The similarity between predicted *R* ^2^ and the adjusted *R* ^2^ confirms the adequacy of the model to predict the response (Table 5). Lower value of coefficient of variation depicts greater reliability of the experiments and thus, our value of 2.78 % indicates that the model is reliable. The “Lack of fit *F* value” of 4.82 tells that the “Lack of fit” was not significant and hence confirming that the model was fit. Adequate precision is an indicator of signal-to-noise ratio and should be greater than 4.0 and our ratio of 88.825 depicts a satisfactory signal. Further, it is extremely important for model to give an adequate fit, which otherwise can lead to prediction of false results. In our case, the adequate fit was satisfied as the normality assumption plot deduced along a straight line giving satisfactory approximation to the test (Fig. 2).

#### Interaction analysis among variables

**Fig. 3:**
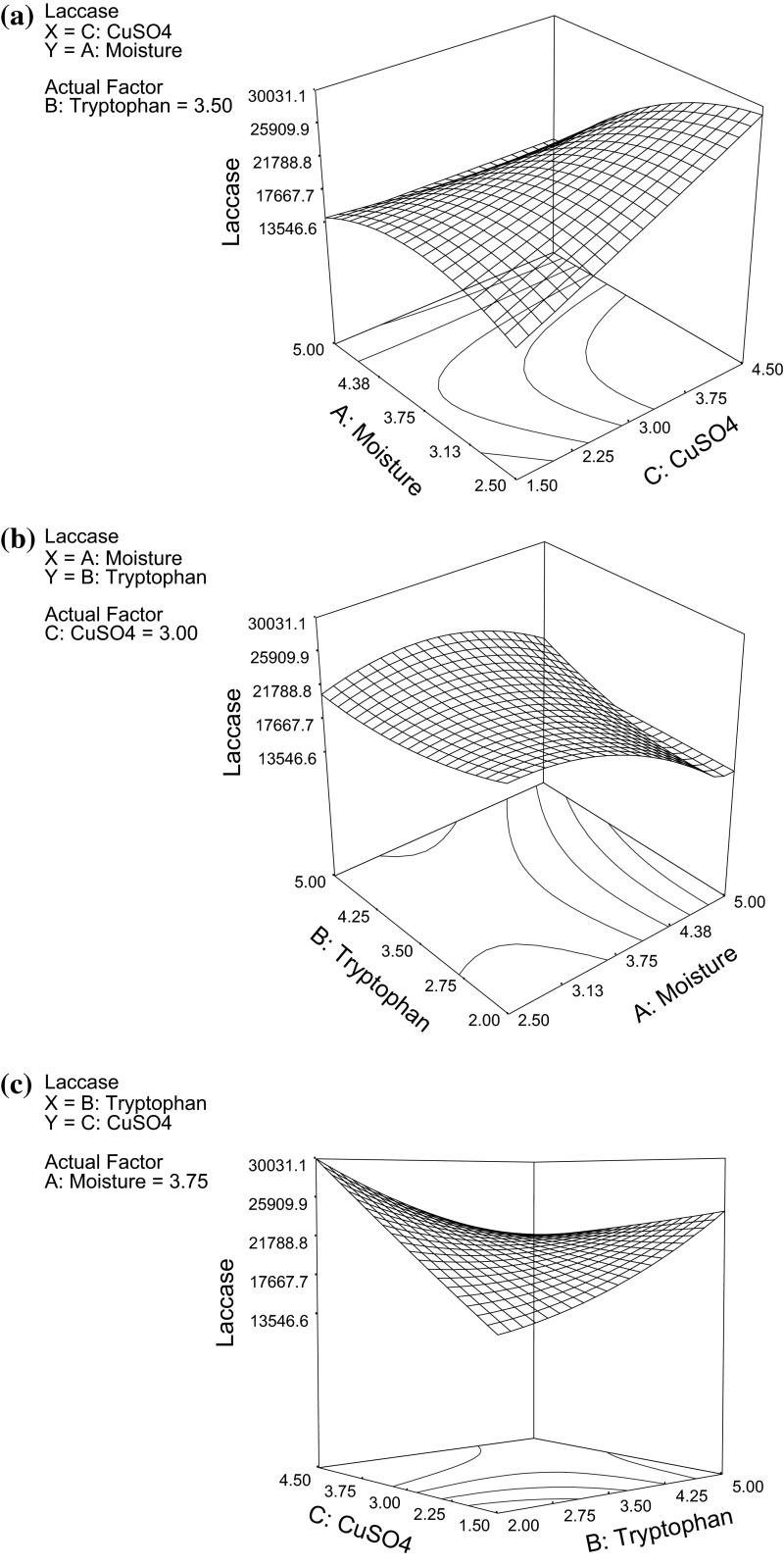
**a** Response surface plot of laccase production as a function of copper and moisture at fixed tryptophan concentration of 3.5 %w/w. **b** Response surface plot of laccase production as a function of moisture and tryptophan at fixed copper concentration of 3.0 mM. **c** Response surface plot of laccase production as a function of tryptophan and copper at fixed substrate to moisture ratio of 1:3.75

The three-dimensional (3-D) response surface plots of laccase production based on the model were generated for the pair-wise combination of the three factors while keeping the other one at its O-level (Fig. 3a–c). The response surface finds out the optimum level of the selected factors for maximum response and also finds a desirable location in the design space. Figure 3a shows effect of interaction between copper and moisture on laccase production, while Fig. 3b depicts interactive effect of tryptophan and moisture on laccase production. It was observed that with increasing initial substrate to moisture ratio from 1:1.65 to 1:2.5 (Fig. 3a, b), a considerable increase in enzyme production was observed from *Ganoderma* sp. rckk-02. While, any further increase in moisture content resulted in a substantial decrease in laccase production. This is because at increased moisture levels, availability of the additives (copper and tryptophan in this case) also increase in the solid medium (wheat bran), resulting in enhanced laccase production. Moreover, at a fixed volume of substrate, increase in water content reduces the air content of the substrate which, in turn affects microbial growth (Battan et al. 2007). While, at lower water levels, the decomposition rate of total organic matter decreases, thereby affecting enzyme production (Raimbault 1998). Patel and co-workers (Patel 2009) have also reported similar effect of moisture on laccase production. Figure 3c shows interactive effect of copper and tryptophan on laccase production. It was observed from the response curve that laccase production increased significantly on increasing copper and tryptophan concentration from their O-level. This suggests that some positive interaction between copper and tryptophan resulted in increased laccase production from *Ganoderma* sp. rckk-02. Further, the large value of copper ion concentration shows its inductive effect on laccase production from the white-rot fungus. Moreover, the laccase titres obtained in the present work are much higher than that reported by other workers from white-rot fungi (Table 6), which could be due to supplementation of high concentration of copper to the cultures of *Ganoderma* sp. rckk-02. Many researchers have shown increased laccase production in presence of copper (Sadhasivam et al. 2008; Santo et al. 2012; Daassi et al. 2013). However, some reports have also shown that high concentrations of copper have detrimental effect on fungal growth and thus on laccase activity. Lorenzo et al. (2005) observed that copper at 20 mM concentration inhibited laccase production from *Trametes versicolor* up to 40 %. While, Gnanamani et al. (2006) found that 30 mM copper ion enhanced laccase production by 3.5-fold from *P. chrysosporium*, suggesting that sensitivity to copper varies with the fungal species, acting as inducer for some species and inhibitor for other, also depending on other culture conditions (Gnanamani et al. 2006). In our work, addition of tryptophan, which acts as nitrogen source might also have inhibited the detrimental effect of high concentration of copper (>2.0 mM) on the fungus. Mishra and Kumar (2007) also observed that high concentrations of copper are not inhibitory to the growth of *A. nidulan*s in presence of cyanobacterial biomass which acts as N-supplement to the basic substrate. Diwaniyan et al. (2011) also observed increased laccase production due to positive interaction between copper and tryptophan.

**Table 6:** Comparison of laccase production under SSF by *Ganoderma* sp. rckk-02 with other fungi

Fungus	Substrate	Laccase activity (U/gds)	References
*P. cinnabarinus*	Sugarcane bagasse	90	Meza et al. (2005)
*P. sanguineus*	Sago hampas	46.5	Vikineswary et al. (2006)
*Ganoderma* sp.	Wheat bran	10,050	Revankar and Lele (2006)
*G. lucidum*	Wheat bran	2,540	Murugesan et al. (2007)
*T. trogii*	Poplar wood	901	Levin et al. (2008)
*P. ostreatus*	Wheat bran	14,189	Patel et al. (2009)
*P. chrysosporium*	a) Brewery waste	738	Gassara et al. (2010)
	b) Pomace	719	
	c) Pulp and paper industry sludge	308	
	d) Fishery waste	94	
*T. trogii*	Soybean cake	219	Zeng et al. (2011)
*T. versicolor*	Brewer’s spent grain	13,506	Dhillon et al. (2012)
*C. caperata* RCK2011	Wheat bran	1,576.13	Nandal et al. (2013)
*Ganoderma* sp. rckk-02	Wheat bran	37,423	Present work

### Decolorization of malachite green by laccase from *Ganoderma* sp. rckk-02

#### Effect of mediator on decolorization of malachite green by laccase

The crude laccase from *Ganoderma s*p. rckk-02 could decolorize 70 % of malachite green within 36 h in presence of the mediator, HBT (1 mM) while, no decolorization was observed in its absence. This is because the redox potential of fungal laccases ranges from 0.5 to 0.8 V, allowing direct degradation of only low-redox potential phenolic compounds. However, in presence of redox mediators which transfer electrons between laccase and the substrate molecule, even non-phenolic compounds with higher redox potential present in the substrate molecule can be oxidized by laccase (Camarero et al. 2005).

#### Effect of enzyme dose on decolorization of malachite green by laccase

**Fig. 4:**
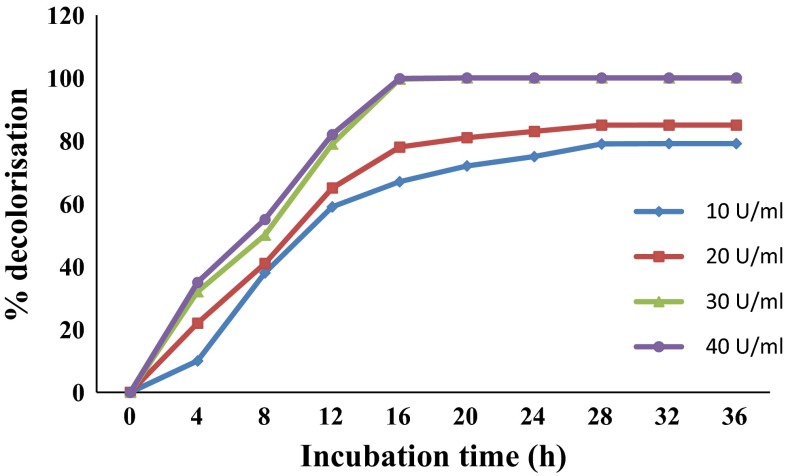
Effect of laccase dose on decolorization of malachite green (100 mg/l)

While studying the effect of enzyme dose on decolorization of 100 mg/l of malachite green, the rate of dye decolorization increased with increase in enzyme dose from 10 to 30 U/ml with almost complete decolorization with 30 U/ml of the enzyme within 16 h (Fig. 4). However, on further increasing the enzyme dose to 40 U/ml, the rate of decolorization did not increase (Fig. 4). Murugesan et al. (2007) also observed similar results with respect to the effect of laccase dose on dye decolorization.

#### Effect of dye concentration on decolorization of malachite green by laccase

**Fig. 5:**
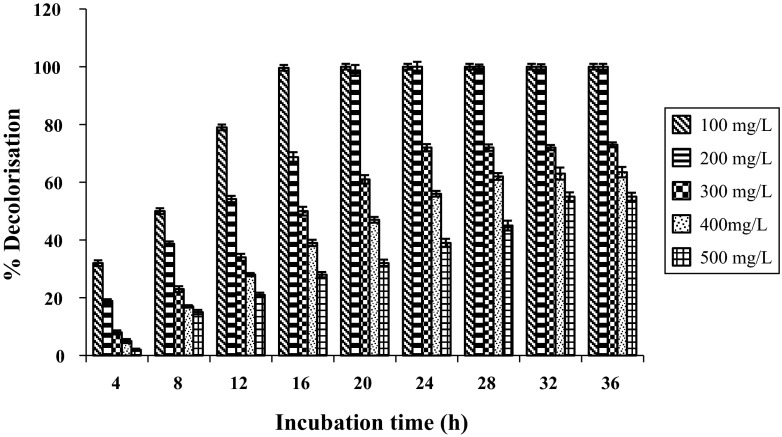
Effect of dye dose (100–500 mg/l) on decolorization of malachite green with 30 U/ml laccase

On studying the effect of dye concentration (100–500 mg/l) on decolorization of malachite green by laccase (30 U/ml), it was observed that on increasing the concentration of dye, percentage of decolorization was slow in the beginning of the experiment, but gradually increased with incubation time up to a point till it became constant (Fig. 5). While, 100 and 200 mg/l of the dye were decolorized completely by laccase in 16 and 20 h, respectively, higher dye concentrations of 300, 400 and 500 mg/l were decolorized to 72, 62 and 55 % in 24, 28 and 32 h, respectively, under similar condition (Fig. 5) Similar results were observed by Satishkumar and co-workers (2010) who inferred that at high concentrations of the dye, efficiency of the enzyme is reduced. The laccase from *Ganoderma* sp. rckk-02 showed better decolorization rate compared to the laccases from other fungi reported (Table 7). Comparable to our results, Yan and co-workers (2014) reported 98 % decolorization of malachite green (200 mg/L) in 20 h by laccase from *T. trogii.* While, there is no report so far on decolorization of malachite green at concentration >200 mg/l (Table 7).

**Table 7:** Comparison of decolorization of malachite green using laccases from different white-rot fungi

Enzyme source	% Decolorization	Dye concentration (mg/L)	Time	References
*Trametes* sp.	80	50	2 h	Maalej-Kammoun et al. (2009)
*T. trogii*	97	7	24 h	Levin et al. (2010)
*T. villosa*	98	7	24 h	Levin et al. (2010)
*C. versicolor*	97	7	24 h	Levin et al. (2010)
*Paraconiothyrium variabile*	60.5	60	15 min	Forootanfar et al. (2011)
Commercial	87.32	25	24 h	Bibi et al. (2011)
*T. versicolor*	85	22 µM	24 h	Grassi et al. (2011)
*P. florida*	96	100	3 h	Balan et al. (2012)
*Trametes trogii*	96	150	8 h	Yan et al. (2014)
	100	100	16 h	
	98	200	20 h	
*Ganoderma* sp. rckk-02	72	300	24 h	Present work
	62	400	28 h	
	55	500	32 h	

### Evaluation of malachite green toxicity towards fungi

Among the five fungal isolates used in the present study, *P. chrysosporium* was most sensitive to malachite green with no mycelial growth at any dye concentration (Table 8). While, other fungi could grow in presence of all dye concentrations, although the growth was very less compared to the control (Table 8). Many other workers have also shown toxicity of malachite green towards fungi (Maalej-Kammoun et al. 2009; Papinutti et al. 2006; Sathishkumar et al. 2010) species namely, *P. chrysosporium* and *Trametes* sp. Therefore, the presence of this dye in water streams will affect the growth of marine fungi as well. Marine fungi are ecologically, morphologically and physiologically important intermediaries of energy flow between plant detritus and marine fauna (Maira and Sridhar 2006). By white-rot or soft-rot decay, these fungi cause more extensive decay of wood in marine habitats than bacteria (Nambiar et al. 2008). However, all these fungal isolates tested, *C. caperata* RCK 2011 (Nandal et al. 2013), *Crinipellis* sp. RCK-1 (Diwaniyan et al. 2011), *Ganoderma* sp. rckk-02 (Sharma et al. 2013), *P. cinnabarinus* (Eggert et al. 1997) and *P. chrysosporium* (Srinivasan et al. 1995), are known producers of laccase. Nevertheless, in presence of dye, the growth of the isolates was retarded and hence laccase was not produced in sufficient amounts for dye decolorization. The difference in growth retardation patterns of the fungal cultures is due to different growth phases in which laccase is produced by each isolate (Papinutti and Forchiassin, 2004). The present work also suggests that removal of malachite green from water bodies is not possible using whole fungal cells as a possible remediation strategy. Therefore, the use of isolated laccase is the best alternative route for elimination of malachite green from water bodies.

**Table 8:** Radial growth of fungal isolates on MEA supplemented with laccase-treated and -untreated malachite green

Fungus	Malachite green concentration (mg/L)	Diameter (cm)	
		MEA supplemented with untreated dye	MEA supplemented with laccase-treated dye
*Crinipellis* sp. RCK-1	100	5.2 ± 0.2	7.5 ± 0.5
	200	4.5 ± 0.3	7.2 ± 0.5
	300	4.0 ± 0.2	4.8 ± 0.4
	400	3.4 ± 0.2	4.0 ± 0.3
	500	3.0 ± 0.3	3.6 ± 0.2
	Control^a^	8.0 ± 0.5	
*C. caperata* RCK 2011	100	1.5 ± 0.2	4.0 ± 0.3
	200	1.0 ± 0.1	3.6 ± 0.3
	300	0.7 ± 0.1	1.1 ± 0.2
	400	0.4 ± 0.02	0.8 ± 0.3
	500	0.1 ± 0.01	0.5 ± 0.1
	Control^a^	4.5 ± 0.2	
*Ganoderma* sp. rckk-02	100	1.3 ± 0.4	3.6 ± 0.3
	200	1.0 ± 0.2	3.1 ± 0.3
	300	0.7 ± 0.1	1.2 ± 0.2
	400	0.5 ± 0.05	1.0 ± 0.1
	500	0.2 ± 0.01	0.5 ± 0.07
	Control^a^	4.0 ± 0.3	
*P. chrysosporium* K3	100	–	7.4 ± 0.3
	200	–	6.9 ± 0.4
	300	–	–
	400	–	–
	500	–	–
	Control^a^	8.0 ± 0.5	
*P. cinnabarinus* PB	100	1.0 ± 0.1	6.1 ± 0.2
	200	0.5 ± 0.03	5.6 ± 0.3
	300	0.3 ± 0.01	0.8 ± 0.2
	400	0.1 ± 0.01	0.6 ± 0.1
	500	–	0.4 ± 0.05
	Control^a^	6.5 ± 0.5	

^a^Control growth of fungus on MEA without the addition of any dye

### Fungal viability on decolorized malachite green

The radial growth of fungal mycelium in MEA containing laccase-treated malachite green increased compared to the growth in culture media containing untreated dye (Table 8). Therefore, it can be concluded that decolorization of malachite green by laccase from *Ganoderma* sp. rckk-02 also reduces its toxicity, making it amenable for fungal growth. It was observed that the growth of all the isolates on MEA supplemented with laccase-treated dyes at 100 and 200 mg/L concentrations was similar to that of the control (Table 8). This indicates that reduced toxicity is associated with the degree of color removal from the dye. The pre-treated dyes at these concentrations get almost completely decolorized by laccase (Fig. 4). While, at dye concentrations of more than 200 mg/l, growth was retarded on MEA amended with pre-treated dyes compared to the control, but more than that on media supplemented with untreated dyes (Table 8). This is because at higher dye concentrations, laccase does not remove malachite green completely (72, 62 and 55 % of 300, 400 and 500 mg/l of malachite green) and hence the residual dye in the laccase-treated dye solutions inhibits fungal growth to some extent. Maalej-Kammoun et al. (2009) also showed removal of toxicity of malachite green against *P. chrysosporium* and *Trametes* sp. after laccase treatment. While, Satishkumar et al. (2010) reported reduced phytotoxicity of malachite green after laccase treatment.

## Conclusion

The decolorization of malachite green by laccase from *Ganoderma* sp. rckk-02 could be very advantageous for application of the enzyme in treatment of textile effluents and fish farms. The statistical methods used for optimizing culture conditions helped in enhancing laccase production by several folds under SSF. Higher productions achieved will lead to reduced cost of the enzyme making its commercial viability for bioremediation strategies possible.
